# From Training to Organizational Behavior: A Mediation Model through Absorptive and Innovative Capacities

**DOI:** 10.3389/fpsyg.2017.01532

**Published:** 2017-09-15

**Authors:** Benito Yáñez-Araque, Felipe Hernández-Perlines, Juan Moreno-Garcia

**Affiliations:** ^1^Department of Business Administration, University of Castilla-La Mancha Toledo, Spain; ^2^Department of Information Systems and Technologies, University of Castilla-La Mancha Toledo, Spain

**Keywords:** absorptive capacity, fsQCA, innovation, organizational performance, PLS-SEM, training

## Abstract

The training of human resources improves business performance: myth or reality? While the literature has extensively addressed this issue, the transfer that occurs from training to performance still remains unresolved. The present study suggests an empirical solution to this gap, through a multiple mediation model of dynamic capabilities. Accordingly, the study makes a major contribution to the effectiveness of an organizational-level training: the “true” relationship between training and performance is mediated by absorptive and innovative capacities. It is difficult from training to directly affect the results: it must be done through a chain of intermediate variables. Training can be argued to be indirectly related to performance, through absorptive capacity in the first place, and innovative capacity in the second, sequentially in this order (three-path mediated effect). Of all immediate relationships received by performance, its explained variance is achieved partly via absorptive capacity and partly via innovation. The direct relationship through training is not significant and only explains a small percentage of the variance in performance. These results have been corroborated by combining two methods of analysis: PLS-SEM and fsQCA, using data from an online survey. This dual methodology in the study of the same phenomenon allows overcoming the limitations of each method, which would not have been possible with a single methodological approach, and confirming the findings obtained by any of them.

## 1. Introduction

Transfer of training refers to the degree to which learned skills and behaviors from the training environment are applied, generalized and maintained in the working environment (Baldwin and Ford, 1988). To determine whether training produces real benefits we must investigate the effects of training on the organizational performance (Tung-Chun, 2001). There is a number of studies examining the positive effects of training on organizational performance (Bartel, 1994, 2000; Barrett and O'Connell, 2001; Aragón-Sánchez et al., 2003; Dolan et al., 2005; Ng, 2005; Barba et al., 2014; Úbeda-García et al., 2014). However, the existence of an explanatory gap in that relationship (Baldwin and Ford, 1988; Aragón and Valle, 2013; Barba et al., 2014; Saks and Burke-Smalley, 2014) together with the scarcity of empirical research on the subject, especially at the level of organizational analysis, have often been criticized (Tharenou et al., 2007).

There is a level gap in the training literature in which, although a goal of the training is to enhance organizational effectiveness, the models, methods, and tools of training focus on the individual level (Kozlowski et al., 2000). Summaries of the training effectiveness literature appear to be limited to the periodic narrative Annual Reviews (Arthur et al., 2003). Lastly, few empirical studies examine the specificities of the models of absorptive capacity, whether it intervenes to translate the different sources of external knowledge flow into realized benefits, such as innovation. In addition, most studies consider innovation as the only outcome of absorptive capacity (Kostopoulos et al., 2011).

In relation to these criticisms, the present study aims to fill a gap in the literature by shedding light on how training is transferred to performance in the unit of analysis at the organizational level and an empirical manner. To do so, the approach of dynamic capabilities is suggested, as an appropriate conceptual basis for determining the connection between training and business performance. This connection is revealed through absorptive and innovative capacities, which play a mediating role. In other words, the study tested whether the dynamic capabilities of absorption and innovation sequentially mediate the relationship between training and business performance.

Another strength of the study is the methodological triangulation combining two different research methods: PLS-SEM and fsQCA, which allows to complement one another and validate a theory through hypothesis testing (Vasilachis de Gialdino, 1992; Bericat, 1998; Lozano, 2010).

Firstly, modeling was used through PLS-SEM (Partial Least Squares Structural Equation Modeling). This method is suitable for the analysis of mediating variables. Secondly, the fuzzy-set qualitative comparative analysis method (fsQCA) was used to complement the information obtained from the former method.

This dual analysis is one of the study's contributions: combining and integrating two methods, thus showing the value of fsQCA against PLS-SEM and overcoming the limitations of the latter when used by itself.

The second major contribution of the study is an important finding for firms, executives and managers: training does not yield benefits in organizational performance unless absorptive capacity in the first place, and innovative capacity in the second, mediate this relationship.

After this introduction, the next section reviews the literature on training, dynamic capabilities of absorption and innovation, and business performance. Next, the research methodology is described, with special emphasis on the two methods of analysis. Finally, research results are presented, along with a discussion of key findings.

## 2. Theoretical background and research hypotheses

### 2.1. Training, performance, and dynamic capabilities

The theory of dynamic capabilities has some aspects related to its conceptualization that seem not to be completely clear, especially in relation to the factors that compose them. Teece (2007) proposed a new model to explain the dynamic capabilities concept detailing some factors that influence their development. This model is formed by some elements that belong either to the absorptive capacity (e.g., learning activities and skill accumulation, processes to identify target market segments, and so on) or the innovative capacity concept (e.g., processes to direct internal R&D and select new technologies, delineating the customer solution and the business model, etc.).

In the literature, we can find up to four main components of the dynamic capacities that, together, explain the mechanisms by which the advantages of internal resources are linked to the competitive advantage based on the external market of the companies: the adaptative capacity or flexibility, the absorptive capacity and the innovative capacity, which could be correlated (Wang and Ahmed, 2007); also, authors as Zollo and Winter (2002) consider in addition the learning capacity. Therefore, within this framework of dynamic capacities are located the absorptive capacity and the innovative capacity, together with flexibility and learning. However, some authors argue that the first two appear to be true components of the dynamic capabilities given that taking into account the adaptative capacity would be a tautological error since the definition of adaptative capacity is implicitly given in the definition of absorptive capacity (Teece, 2007; de Castro et al., 2009). Similarly, differentiating learning capacity from dynamic capacities when the absorptive capacity is taken into account seems to be another tautological error (de Castro et al., 2009), since the absorptive capacity process carries implicit the learning capacity (Cohen and Levinthal, 1990; Lane et al., 2001) or, in any case, the learning capacity would emanate from the absorptive capacity, being two similar and interrelated processes and even “interchangeable” terms (Cohen and Levinthal, 1989). For these reasons, a large number of researches have focused their interest in the dynamic capabilities of absorptive capacity and innovation.

In previous studies, authors like Brettel et al. (2011); Todorova and Durisin (2007); and Van den Bosch et al. (1999) suggest the importance of absorptive capacity (hereafter ACAP) to improve performance and build competitive advantages.

Hernández-Perlines and Yáñez-Araque (2015) provide a theoretical overview of the interlinking relationships between complementary concepts of training, as a starting point for the Intellectual Capital and other multiple interactions among Knowledge Management, Organizational Learning, ACAP, training and performance. Thus, according to these authors, training has an impact on results through the ACAP process. They conclude that ACAP is the necessary mechanism to turn training into tangible benefits.

The literature recognizes that both ACAP and innovation are two key dynamic capabilities in obtaining competitive advantages. What is more, several authors have studied the interrelationships between these two dynamic capabilities, confirming that firms with a higher level of ACAP invest more in Research and Development (R&D) and can therefore perform more innovations (Tsai, 2001; Mei and Nie, 2007; Aljanabi et al., 2014). “The ability of a firm to recognize the value of new external know-how and assimilate it (ACAP) is critical to its innovative capabilities” (Montazemi et al., 2012, p. 36).

In other words, absorptive and innovative capabilities are so closely related that, should a firm not have the necessary absorptive capacity, it will not get any benefit from innovation (Kostopoulos et al., 2011). Therefore, if—as Hernández-Perlines and Yáñez-Araque (2015) suggest in their seminal work—training only translates into tangible results through ACAP, the question arises whether also innovation plays a mediating role in obtaining revenue from training activities.

In the following sections, interrelations among the main concepts of this study will be specified based on the literature review, which will help state the research hypotheses.

### 2.2. Relationship between training and business performance

Most studies examining the relationship between training and performance (Wright et al., 1994; Kamoche, 1996; Mueller, 1996; Barney and Wright, 1998; Bassi et al., 1998; Lee and Yang, 2000; Hitt et al., 2001; Tung-Chun, 2001; Ordonez de Pablos, 2004; Tharenou et al., 2007; Saks and Burke-Smalley, 2014; Úbeda-García et al., 2014) focus on the role training plays in the development of human capital (Schultz, 1961; Becker, 1964; Fahy, 2000; Úbeda-García, 2005) and organizational knowledge (Alavi and Leidner, 2001; Bollinger and Smith, 2001).

The literature has extensively analyzed the positive impact of training on organizational results. There are studies linking training and benefits, training and productivity, training and competitive advantage; and studies linking training to other aspects of business results (Marin-Diaz et al., 2014).

According to the above ideas:

**H1**. Training positively affects organizational performance.

### 2.3. The mediating role of absorptive capacity

Based on the above, firms could believe that the simple introduction of training is enough to improve their performance. However, without absorptive capacity, firms would not obtain the expected benefits from training. Some early studies in literature already approached the idea that absorptive capacity helps to explain how the transformation process of training occurs in organizational performance (Spence, 1973; Taubman and Wales, 1973), thus being a mediating variable. In this sense, ACAP is revealed as one of the key elements that strengthens the relationship between training and business results. The meaningful learning theory helps to better understand how training and ACAP are closely linked. Meaningful learning is the type of learning by which a student relates the new information to the one he already has, adjusting and reconstructing both information in this process (Ausubel, 1968). Meaningful learning is what leads to transference. Similarly, the dynamics of ACAP have a cumulative development, in the same manner as in the process of meaningful learning. Experience or performance in a learning task can influence and improve performance in a subsequent learning task (Ellis, 1965). Van den Bosch et al. (1999) study the determinants of ACAP, being the level of prior related knowledge one of its determinants. Cohen and Levinthal (1990) propose that ACAP affects a firm's expectation formation, allowing the company to more fully predict the nature and commercial potential of technological advances (*path dependency* of ACAP). As a direct consequence, by similarity with cognitive structures literature, they conclude that the accumulation of ACAP in a given period allows a more efficient accumulation in the next one. These authors see ACAP as a self-reinforcing process, through which ACAP enriches the learning capacity of the firm, and vice versa. They define the absorptive capacity of a firm as “prior related knowledge confers an ability to recognize the value of new information, assimilate it, and apply it to commercial ends” (p. 128). Finally, they consider a capacity to absorb knowledge requires a pertinent formal training.

Zahra and George (2002) present a fresh conceptualization of ACAP as a multidimensional construct representing the firms dynamic capacity to create and use knowledge relating to the firms ability to compete. According to them, ACAP comprises four dimensions split into two capacities: potential ACAP (acquisition and assimilation) and realized ACAP (transformation and exploitation).

By definition moderator effect happens if the intensity or direction of the relationship between a dependent variable and an independent one is affected by another independent variable (Hair et al., 2000) which has the ability of distorting this relation. The moderator variables are always independent variables. Thus, whereas a priori, in order to have a moderator effect the moderator should not be related neither with the independent nor the dependent variable (it only affects their relationship), in the mediation, the mediator is explanatory (mechanism that shows how or why the outcome is generated from the input) and it is necessarily correlated with both the dependent and independent variables. The mediator is internalized as part of the process that takes place between the independent variable and the dependent variable. To demonstrate mediation, one must establish strong relations between the independent and the mediating variable and, in turn, between the mediating variable and some dependent variable (Baron and Kenny, 1986). In this way, from the theoretical point of view, we justify a mediating effect if there are some references in the literature that relate our mediating variable to the independent variable (causally antecedent of the mediator), on the one hand, and, on the other hand, with the output variable (the mediator as antecedent of the latter). This is what happens in our case: ACAP is related to both training (training is the causal antecedent of ACAP) and to organizational performance (ACAP is the causal antecedent of organizational performance). This is what this study does, which allows you to state the following hypothesis:

**H2**. ACAP positively mediates the relationship between human resources training and organizational performance.

Mathieu and Taylor (2006) define mediation in terms of understanding how some antecedent variable (X = training) affects a criterion variable (Y = organizational performance) as transmitted through a mediating variable (M = ACAP). Smith (1982) proposed an ingenious solution to the problem of feedback in mediational chains. His method involves the independent variable to cause the mediator, the mediator to cause the dependent variable and the dependent variable not to cause the mediator. Models of this type are estimated by two stages (Baron and Kenny, 1986). This logic is in line with the so-called causal steps strategy or approach (Preacher and Hayes, 2008; Taylor et al., 2008). Accordingly, mediation hypotheses must split into two sub-hypotheses of relations that are sequentially taken in pairs (X → M; M → Y).

Indeed, the origins of the ACAP concept had already reported the first connection between training and ACAP: Cohen and Levinthal (1990) argued that the concept of absorptive capacity can best be developed through an examination of the cognitive structures that underlie learning.

Acquiring new external knowledge is the antecedent of ACAP (Van den Bosch et al., 1999; Zahra and George, 2002), whereas training is the input of ACAP (Cohen and Levinthal, 1990; Yahya and Goh, 2002). In this sense, training generates a certain type of knowledge flow that should be absorbed and processed by the firm's ACAP in order to obtain results. In other words, training represents a particular source of “raw material” for knowledge; whereas ACAP is the way this raw material is managed, like a filter mechanism or sieve through which knowledge emanating from training is transformed into results for firms. Or put another way:

**H2a**. Training positively affects ACAP.

Several studies focus on how business performance results from ACAP. In fact, empirical findings reveal a significant positive relationship between ACAP and business performance (Mowery et al., 1996; Mukherjee et al., 2000; Lane et al., 2001, 2006; Tsai, 2001; Zahra and George, 2002; Jansen et al., 2003, 2005; Todorova and Durisin, 2007; Bergh and Lim, 2008; Yeoh, 2009). Therefore, this study suggests the following sub-hypothesis:

**H2b**. ACAP exerts a positive influence on organizational performance.

**Figure 1:**
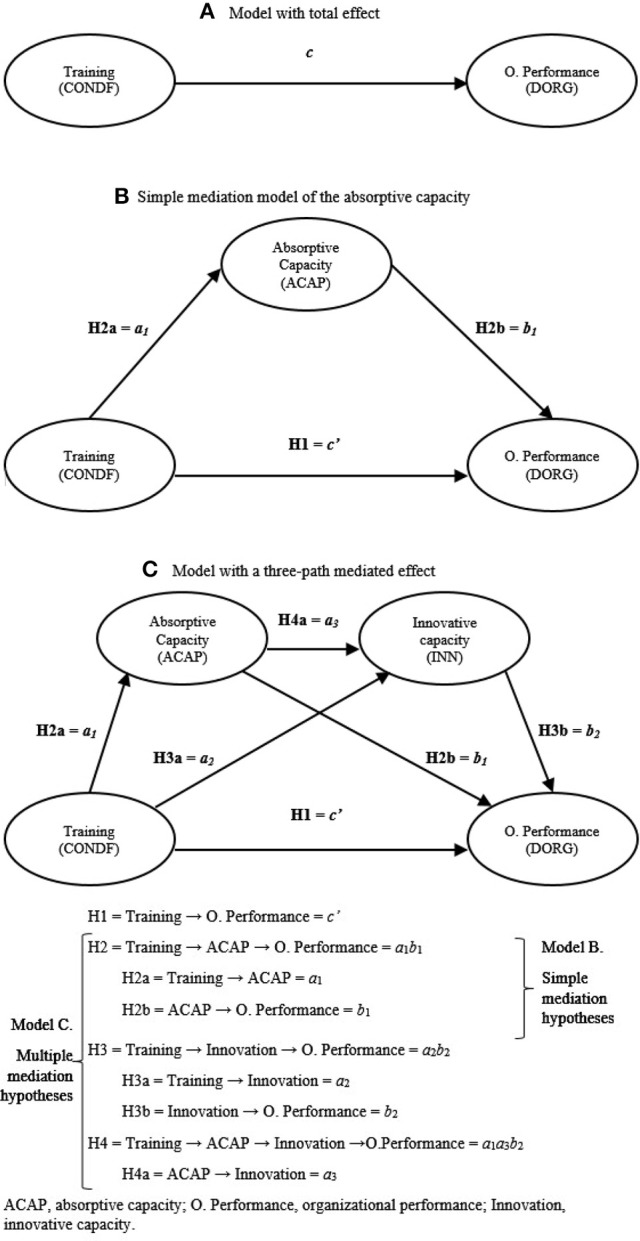
Multiple mediating conceptual model. **(A)** Model with total effect. **(B)** Simple mediation model of the absorptive capacity. **(C)** Model with a three-path mediated effect.

According to the above ideas, Figures 1A,B represents the direct or original model (Model A: model with total effect), i.e., the relationship between training and performance, which is mediated by absorptive capacity (Model B: model of simple mediation of absorptive capacity).

### 2.4. The mediating role of innovative capacity

This study has used the definition of innovation provided by Carnegie et al. (1993: p. 3) “something that is new or improved and done by the enterprise to create significantly added value either directly for the company or indirectly for its customers.”

While this definition—which was also adopted by Prajogo and Sohal (2006)—represents a broad view of innovation which contrasts with the specific and traditional concept based on R&D, it suits the purpose of this study.

There are some studies that positively consider training as a determinant of innovative capacity (Greenhalgh and Mavrotas, 1994; Baldwin and Johnson, 1995; Frazis et al., 2000; Huergo, 2002; Beugelsdijk, 2008; Bauernschuster et al., 2009). However, these budding studies do not have enough empirical evidence to address this issue in depth. Thus, an interesting contribution would be to better clarify the relationship between the above two business strategies (García Espejo, 2008; Dostie, 2014).

Much more addressed and demonstrated is the relationship between innovation and performance, where high levels of innovation are generally associated with high levels of performance (Armour and Teece, 1978; Rosenberg, 1982, 1994; Damanpour et al., 1989; Subramanian and Nilakanta, 1996; Crépon et al., 1998; Klomp and Van Leeuwen, 2001; Lööf and Heshmati, 2006; Rosenbusch et al., 2011).

There are even authors who have suggested the link between training and performance to be partially established through innovation (Laplagne and Bensted, 1999; Dostie, 2014).

Hence the following set of hypotheses:

**H3**. The relationship between training and organizational performance is positively mediated by innovative capacity.
**H3a**. Training exerts a positive influence on innovative capacity.
**H3b**. Innovative capacity exerts a positive influence on organizational performance.

Hernández-Perlines et al. (2016) argue that ACAP is a mediator in the relationship between training and performance. In other words, ACAP completely absorbs the effect of training and transforms it into a better performance. However, taking into account earlier views and literature, besides the direct effect exerted by ACAP on performance, there could also be an indirect effect, generated by the inclusion of innovative capacity as a second variable mediator of training and by the inclusion of a part of the effect of ACAP on performance. In theory, the inclusion of this second dynamic capacity (innovation) in the model should explain the greater variability in performance (dependent variable) as compared to the simple mediation model of ACAP.

Therefore, this study suggests a multiple mediation model to explain how training is transferred to performance through not only ACAP, but also innovative capacity, where ACAP would sequentially precede innovation (Cepeda-Carrion et al., 2012). In fact, some authors have already studied this sequence, demonstrating that ACAP and similar processes of absorption of knowledge positively influence innovation; and the latter, in turn, has a positive effect on performance (Tsai, 2001; Darroch, 2005; Wang and Wang, 2012).

In view of the theory and empirical evidence aforementioned, training is related to performance in the first place, through ACAP, and in the second, through innovative capacity. The integration of these two mediation models (ACAP and innovation) generates a model with a three-path mediated effect (Figure 1C, Model C) (Taylor et al., 2008; Hayes, 2009). Therefore:

**H4**. The dynamic capabilities of absorption and innovation sequentially mediate the relationship between training and organizational performance.

This mediation hypothesis supports the breakdown in H2a and H3b sub-hypotheses listed above and poses a new sub-relationship that has been widely advocated in the literature (Orlay, 1993; Tsai, 2001; Matthews, 2002; Mei and Nie, 2007; Kostopoulos et al., 2011; Cepeda-Carrion et al., 2012; Aljanabi et al., 2014) namely:

**H4a**. Absorptive capacity exerts a positive influence on innovative capacity.

## 3. Research methodology

### 3.1. Selection and measurement of variables

This study analyzes a limited number of variables from a broader research project. All selected variables were measured based on scales validated in previous studies. In particular, the following variables have been considered according to the research objectives (the complete list of the measurement items can be found in the **Appendix**).

#### 3.1.1. Training (CONDF)

Independent variable or predictor. The study measured training (reflective first-order latent variable in PLS-SEM method) using a validated 5-item scale suggested by Castañeda and Fernández (2007). For the purpose of the study, the Likert scale is adapted to 7 points or response levels for all indicators, to collect data on whether the firm fosters the necessary training conditions to trigger ACAP. This is why this scale is very suitable for the relationships suggested in this research, and also because it represents a general measure of the importance that firms attach to the training of employees.

#### 3.1.2. Organizational performance (DORG)

Dependent or endogenous variable. This study measured this criterion variable using the scale validated by Camisón and Villar-López (2010), which has two dimensions: economic performance (five items) and satisfaction performance (four items), all of which used a 7-point Likert-type scale. In the PLS-SEM analysis, DORG is a molecular second-order latent construct whose two first-order dimensions are reflective. The measurement of these first-order dimensions used reflective indicators. The selection of this scale fits the purpose of this study, as training is often considered by the literature as a source of competitive advantage and, therefore, likely to achieve a higher performance. Moreover, the scale connects to business performance in terms of obtaining sustainable sources of competitive advantage (Camisón and Villar-López, 2011), collecting aspects of business performance as compared to competition.

#### 3.1.3. Absorptive capacity (ACAP)

Mediator Variable. The study considers ACAP as a multidimensional variable consistent with the proposal by Cohen and Levinthal (1990) or Lane et al. (2006). The measurement of ACAP used a four-dimension scale validated by Flatten et al. (2011), who evaluate the extent to which the firm engages in knowledge-acquisition activities (acquisition, 3 items), assimilates acquired information with existing knowledge (assimilation, 4 items), transforms recently adapted knowledge (transformation, 4 items) and commercially exploits knowledge transformed into competitive advantage (exploitation, 3 items). All items use seven-point Likert-type response scales. For PLS-SEM, ACAP is a reflective second-order construct with four reflective first-order dimensions. Thus, although each dimension comprises different facets, the assumption is that all four dimensions should be present for the firm to possess genuine absorptive capacity.

#### 3.1.4. Innovative capacity (INN)

Mediator Variable. The study adopts the construct suggested by Prajogo and Sohal (2006), as it holistically captures all aspects and criteria for innovative performance covered in previous empirical studies on innovation. Additionally, in order to design the scale, the authors established questions in terms of the firm's comparison with its competitors (as occurs in the scale selected by this study to measure organizational performance). This approach reduces the subjective response bias (Kraft, 1990). The construct is applied on two major areas of innovation: product innovation (measured with 5 items) and process innovation (4 items or indicators). The original Likert scale is adapted to 7 response levels for all indicators. In the PLS-SEM analysis, INN is a reflective second-order latent construct whose two first-order dimensions are also reflective.

#### 3.1.5. Control variables

The control variables relate directly to criteria variables. Control variables are sector (dummy variables), firm size and firm age, introduced on organizational performance, which is the dependent variable of interest.

### 3.2. Sampling and data collection

For both PLS-SEM analysis and fsQCA the sample comprises 112 valid cases. These cases contain no missing values for the variables under study. The primary database is based on a final questionnaire entitled Spain Survey of Training and Dynamic Capabilities of the Firm (STraDyCaF) (www.stradycaf.org), which was pre-tested and improved in an earlier phase of the study. Data collection took place between May and December 2014 via LimeSurvey Version 2.05+. This open-source web application that specializes in creating and distributing questionnaires and managing target populations was used to send participants (senior executives) a personalized email, together with a cover letter introducing the research project. To encourage responses and improve the response rate, an automatic and individualized follow-up of the respondent was scheduled so that 15 days after the initial survey has been sent, an automatic reminder email was sent to respondents who had not responded (Dillman, 2007); likewise, the questionnaire followed a responsive web design, whereby managers could respond using mobile devices, including a text-to-speech assistant similar to CATI (Computer-Assisted Telephone Interviewing) systems. The sample is representative and consists of Spanish firms from the entire country, having 50 or more workers, and operating in any sector except public administration, agriculture, fishing, activities of households and extraterritorial bodies. Therefore, the analysis is multisectorial in order to avoid the biases of sectorial studies for which other research has been criticized (Huselid, 1995). Sample selection used simple random sampling without replacement, extracted from Iberian Balance sheets Analysis System (SABI) database developed by INFORMA D&B in collaboration with Bureau Van Dijk, which allows the handling of general information and annual accounts of Spanish companies and Portuguese ones. The response rate was 7.18%, similar to the average of postal surveys in Spain where there is no strong tradition of collaboration with centers of research in Spain (Very et al., 1997; del Brío et al., 2002; Roca-Puig and Bou-Llusar, 2007). Another reason for the low response rate is the extension of the entire survey (230 variables) along with the difficulty of getting executive staff to spend their time responding it.

Table 1 summarizes the technical specifications of the study.

**Table 1:** Technical data sheet.

Population scope (universe)	Spanish companies with 50 or more employees, in any sector except public administration, agricultural sector and activities of households and extraterritorial organizations and bodies^a^
Geographical scope	All the national territory/Spanish national territory
Sampling unit/ unit of analysis	Firm
Population census^b^	22,013
Sample size/ response rate	112 valid surveys/7.18%
Sampling procedure	Simple random sampling without replacement
Confidence level	95%; *z* = 1.96; *p* = *q* = 0.50; α = 0.05
Sampling error	9.24%
Key respondents	Senior Executives
Date of fieldwork/ data collected	Between May and December 2014

a *The sectors/economic activities excluded correspond to the following CNAE 2009 and NACE Rev. 2 sections: (A) agriculture, forestry, and fishing; (O) public administration and defense, compulsory social security; and (T) activities of households and (U) extraterritorial organizations and bodies*.

b *Source: DIRCE 2014 (Central Business Register, CBR or DIRCE in Spanish, at 1 January 2014)*.

#### 3.2.1. Sample characteristics and key respondent

Non-response bias was assessed. Two groups were examined for key size and sector variables, the surveys received in the first 2 weeks and the surveys responded later. All *t*-test done did not show significant differences between these two groups, suggesting that there was no response bias and it is not a problem for generalization. Table 2 reflects the representative nature of the sample analysed in terms of size and activity sector of the target population, and there are no significant differences.

**Table 2:** Sample characteristics.

	**Population^a^ (%)**	**Sample (%)**
**SECTOR**		
Industry	5,151 (23.4)	27 (24.11)
Construction	1,176 (5.34)	6 (5.36)
Services	6,638 (30.15)	34 (30.36)
Financial and professional	3,843 (17.46)	19 (16.96)
Education, human health and social works, arts, entertainment and recreation private sector	5,205 (23.65)	26 (23.21)
Total	22,013	112
**SIZE**		
Between 50 and 499 employees	20,322 (92,32)	98 (87.5)
More than 499 employees	1,691 (7,68.4)	27 (24.11)

a *Source: DIRCE 2014*.

The sampling error was 9.24%, an acceptable value since it is equal to or less than 10% (Perelló, 2011). The power value (0.8001) was obtained using the statistic tool G^*^Power Version 3.1.9.2 (Faul et al., 2009). It is greater than the cut-off value (0.8). Then, the sample power of the survey is valid, assuming an error value of 0.05 and an effect size of 0.2301, considered as *small* (Cohen, 1988).

When the unit of analysis is the firm, we assume that an individual acts as a qualified informant that provides data on the characteristics of the company (one-person-per-firm). Can the responses of an individual be extrapolated to the whole company? This is assumed by the dominant logic of most studies (Woodside, 2011). To minimize key informant bias (Phillips, 1981) the hierarchical and functional position was controlled. This was done by imposing the restriction that the key respondent must be an appropriate and well-qualified informant: the most senior executive in charge (Osterman, 1994) or the one who had the highest level of information possible on what was being asked and that was related to the processes of knowledge. To ensure this, the survey was sent to key respondents with this profile and the surveys answered by other informants were rejected. Besides, it was assured to respondents that their answers were anonymous and there were no right or wrong answers, and they were encouraged to answer the questions as sincerely as possible. In addition, the survey includes several types of organizational performance measures, and a highly positive and significant correlation were found between them (Podsakoff et al., 2003). The job titles and positional status of the respondents were: CEO/Chief Executive Officer (top executive), 22 (19.64%); Managing Director, 26 (23.21%); Head of Training, 21 (18.75%); Chief Human Resource Officer, 35 (31.25%); other executive staff, 8 (7.14%).

The paper study collected data on both the exogenous and endogenous variables from the same respondents at one point in time and using the same instrument, thus potential common method variance as false internal consistency might be present in the data. We tested for common methods bias to establish that such bias did not distort the data we collected. To do so, we used two approaches. First, we examined the exploratory, unrotated factor analysis to find the results of Harman's single-factor test for all of the first-order constructs using a standard statistical package. The aim of the test is to determine whether a single factor emerges that explains the majority of the variance in the model. If so, then common method bias likely exists on a significant level (Lowry and Gaskin, 2014).

The result of our factor analysis produced 20 distinct factors, the largest of which accounted for 44.60% variance explained by a single factor. This shows that the common method bias was not a major concern in this study (less than 50% cut-off point).

In a second approach, we analyzed the common latent factor (CLF) to capture the common variance among all observed variables in the model. Adding a first-order factor to all observed items in the model and comparing the standardized regression weights from this CFA model to the standardized regression weights of a model without the CLF (Gaskin, 2017), the results show that all the values are similar (the difference is less than 0.2). As such, common method bias was not a major threat in our data set.

### 3.3. Data analysis: combination of methods

As mentioned earlier, this study employs two methods. Firstly, the PLS-SEM method (Partial Least Squares Structural Equation Modeling) is conducted.

Following Lowry and Gaskin (2014) p. 123, first-generation (1G) techniques are statistical methods, such as correlations, regressions, or difference of means tests (e.g., ANOVA or *t*-tests), that are well suited to simple modeling scenarios. However, 1G techniques offer limited modeling capabilities, particularly in terms of causal or complex modeling. The strength of SEM is in modeling. In particular, SEM allows for complex models that include latent (unobserved) variables, chains of effects (mediation), as is the case at hand.

SEM assesses in a single, systematic and comprehensive way (Gefen et al., 2011) the measurement model (relationships between latent variables and their indicators) and the structural model (the part of the entire model that describes relationships between latent variables; these relationships reflect substantive hypotheses based on theoretical considerations).

The holistic analysis that SEM is capable of performing can be carried out via one of two distinct statistical techniques (Barroso et al., 2010):

1. Methods based on the analysis of covariance (factors), i.e., Covariance-Based SEM (CBSEM);
2. Methods based on variance (or components, or composites), i.e., Partial Least Squares (PLS).

The two approaches were designed to achieve different goals. CBSEM focuses on estimating a set of model parameters so that the theoretical covariance matrix implied by the system of structural equations is as close as possible to the empirical covariance matrix observed within the estimation sample (Reinartz et al., 2009). PLS works with blocks of variables (components) and estimates the model parameters by maximizing the variance explained by all the dependent variables (both latent and observed) (Chin, 1998b).

Going back to Lowry and Gaskin (2014), in choosing whether to use PLS or CB-SEM (both are two specialized forms of SEM), one should initially consider whether the research is exploratory (building or testing a new theory) or confirmatory (testing a well-established theory). For exploratory work, PLS should be selected. For confirmatory work, either technique may be used. PLS can provide advantages over 1G techniques and CB-SEM techniques for preliminary theory building. PLS is also especially useful for models that have higher-order constructs (in this study, three of all latent variables are multidimensional second-order constructs). PLS does not need to assume that the dependent variables conform to any particular distributions. As a result, it is robust to violations of multivariate normal distributions, whereas CB-SEM assumes data normality.

With this in mind, PLS is particularly interesting when theory development is in its early stage (Ringle et al., 2005; Gefen et al., 2011), when researchers adopt scales that studies have already checked or validated, when the analysis uses a relatively small sample (Reinartz et al., 2009) and/or non-normal data distributions (Lowry and Gaskin, 2014), or, finally, when models are highly complex (Hair et al., 2014) and with multidimensional constructs.

Other references in this line of research that justify using PLS are very recent advances (e.g., Henseler et al., 2014; Nitzl et al., 2016; Rigdon, 2016; Sarstedt et al., 2016; Nitzl and Chin, 2017).

For all the above reasons, PLS-SEM is suitable in this study. However, PLS-SEM is not free from limitations: causal symmetric relationships, net effects, etc. These limitations are largely attributable to the problems inherent in multiple regression analysis (MRA) and structural equation modeling (SEM) (Woodside, 2013; Skarmeas et al., 2014).

Secondly, the fsQCA method (fuzzy-set Qualitative Comparative Analysis) overcomes the limitations of the first method. This is a useful qualitative method for analyzing social phenomena with small data sets, allowing for a good management of uncertainty (Ragin, 2000, 2008). fsQCA is used for configurational and causal analysis, where different constellations of variables cause different outcomes. For this reason, we utilize fsQCA to make a *post-hoc* analysis to compare the results obtained by PLS. Our approach has been developed in the same way that El Sawy et al. (2010) to show a fsQCA-based configurational analysis. They followed the recommendations of Ragin (Ragin, 2008, Chapter 11). We are trying to demonstrate the influence of each variable in the outcome variable. The complete dataset is used as input to fsQCA to obtain the results.

For the PLS-SEM analysis, the software tool used was SmartPLS 2.0.M3 (Ringle et al., 2005). For the fsQCA analysis, the data analysis tool was FsQCA 2.5 Software (Ragin and Sean, 2014).

### 3.4. Mediation analysis

According to this research model (Figures 1B,C), H2, H3 and H4 represent mediation hypotheses, suggesting how or by what means an independent variable (training) affects a dependent variable (organizational performance) through mediating variables or mediators (dynamic capabilities of absorptive and innovative capacities) (Preacher and Hayes, 2008).

PLS path analysis allows to evaluate mediation models and tests mediation hypotheses, using the bootstrapping method (Hayes, 2009). As a result, the study uses the bootstrapping method to test mediation, i.e., the importance of indirect effects. Bootstrapping is a non-parametric re-sampling procedure for the analysis of both simple and multiple mediation. It imposes no assumptions about the distribution of variables or the distribution of the sample and can be applied to small sample sizes with more confidence. Therefore, this approach is perfectly suited for the PLS-SEM method (Hair et al., 2014). Authors like MacKinnon et al. (2004) have proven that this method leads to higher performance and higher levels of statistical power than traditional ways of testing mediation hypotheses, such as the Sobel test (Sobel, 1982, 1986), which requires a normal distribution of indirect effects in the sample along with other problems that prevent its application with PLS.

Therefore, mediation hypotheses (H2, H3, and H4) and sub-hypotheses deriving from bootstrapping are contrasted by PLS-SEM.

PLS-SEM is applied to each of the two mediation models suggested. Namely:

- Model B: model of simple mediation of absorptive capacity. It tests whether training affects performance through absorptive capacity (mediation hypothesis H2).
- Model C: model with a three-path mediated effect or multiple mediation model (Taylor et al., 2008; Hayes, 2009). To the previous model of simple mediation, this study has added a second mediating variable: innovative capacity. Therefore, the study re-assesses mediation hypothesis H2 in relation to the new multiple mediation model, along with new mediation hypotheses generated by the second mediating variable (H3 and H4), thus testing all mediation hypotheses in the same model.

Finally, with the purpose of ensuring that the introduction of a second mediating variable explains the relationship between training and performance better than the simple mediation model, both models (B and C) are compared to each other.

Figure 1A shows the total effect of training on organizational performance, where c is the path coefficient or beta weight of training on organizational performance. This total effect can be reached through a variety of direct and indirect forces (Hayes, 2009).

Specifically, Figure 1B (Model B) expresses the total effect of training on performance as the sum of direct and indirect effects. The estimation of the latter uses the product of path coefficients in the mediation chain. Thus: $c=c^{\prime }+a_{1}b_{1}$; where the latter is the indirect effect and *c*′ is the direct effect of training on performance (H1) that controls the absorption capacity mediating variable.

Figure 1C (Model C) expresses the total effect of training on performance as the sum of direct and indirect effects. The estimation of the latter also uses the product of path coefficients, but this time for each of the paths in the mediation chain (Alwin and Hauser, 1975). Thus: $c=c^{\prime }+a_{1}b_{1}+a_{2}b_{2}+a_{1}a_{3}b_{2}$; where the last three are the specific indirect effects and the sum of all of them is the total indirect effect (Hayes, 2009), whereas *c*′ is the direct effect of training on performance (H1), which now controls both mediators (absorptive and innovative capacities) (Taylor et al., 2008). The advantage of this approach is that it is capable of isolating the indirect effect of both mediating variables, i.e., absorptive capacity (H2: *a*_1_*b*_1_) and innovative capacity (H3: *a*_2_*b*_2_). Moreover, this method allows to analyze the indirect effects that go through both mediators in a series (H4: *a*_1_*a*_3_*b*_2_) (van Jaarsveld et al., 2010).

## 4. Findings

The results of this study are structured for each of the two mediation models: the simple model (Model B) and the multiple model (Model C: model with a three-path mediated effect), and resulted from applying the two proposed methods. Due to the nature and analysis of mediation, model A is included in mediation models B and C. Results from models A, B, and C are compared using PLS-SEM. Finally, fsQCA method is applied.

Analysis and interpretation of the model using PLS-SEM take place in two stages (Barclay et al., 1995): (1) Analysis of the measurement model (outer model) and (2) Analysis of the structural model (inner model). This sequence ensures that the measurement scales are valid and reliable.

As indicated in the above section discussing measurement of variables, all constructs are reflective, so that all models are applied reflectively. The evaluation of reflective measurement models examine their reliability and validity (Henseler et al., 2009). In particular: item reliability, construct reliability, convergent validity and discriminant validity (Hair et al., 2012), as will be discussed in the following sections.

Evaluation in the structural model assesses sign, magnitude and significance of the relationships between variables (structural path coefficients: β), explained variance of endogenous variables (*R*^2^) and Stone-Geisser test (*Q*2). Moreover, as this is a reflective measurement model (Hair et al., 2012), the study can apply the holistic approach of Goodness-of-Fit (GoF) (Tenenhaus et al., 2005). The path coefficients or beta weights (β) represent the extent to which the predictor variables contribute to the explained variance of the endogenous variables. In other words, they indicate the relative strength of statistical relationships. For path coefficients to be considered significant, they should reach a minimum value of 0.2, and ideally greater than 0.3 (Chin, 1998a). In this sense, Falk and Miller (1992) suggest that, calculating the variance in an endogenous construct explained by another latent variable—such as the absolute value of the product of β explained by its corresponding correlation coefficient between the two variables, suggesting that a predictor variable should explain at least 1.5% of the variance in an endogenous or predicted variable, i.e., the explained variance of an antecedent variable to the endogenous variable—should reach a minimum value of 0.015. For its part, the explained variance of endogenous variables (*R*^2^) determines how much of the variance in endogenous variables can be explained by the constructs that predict them. Falk and Miller note that *R*^2^ should be greater than or equal to 0.1. Chin (1998b) classifies *R*^2^ levels as weak (*R*^2^:0.19), moderate (*R*^2^:0.33) and substantial (*R*^2^:0.67). The next indicator is the cross-validated redundancy index or Stone-Geisser *Q*2 blindfolding algorithm (Geisser, 1974, 1975; Stone, 1974) for endogenous (reflective) constructs obtained using the blindfolding procedure (Chin, 2010). This procedure only applies to endogenous (reflective) variables and endogenous constructs of a single item (Hair et al., 2014). *Q*2 indicates the predictive relevance of the path or structural model (Chin, 1998a). In the structural model, *Q*2 values that are greater than zero for a certain latent endogenous reflective variable indicate predictive relevance in the path model for that particular construct, while *Q*2 values less than 0 suggest lack of predictive relevance in the model for that particular variable (Hair et al., 2014).

In order to evaluate the statistical significance of path coefficients, standard errors and t-statistics (pseudo-parametric test) are generated from the bootstrapping procedure (5,000 subsamples) (Hair et al., 2011). Similarly, the non-parametric approach (not based on any distribution) reports bootstrap confidence intervals of beta weights and indirect effects for mediation hypotheses. According to Henseler et al. (2009): if the confidence interval estimated for the path coefficient does not contain zero, it means that the estimated path coefficient is significantly different from zero, where the percentage (confidence level) is used to calculate confidence intervals. Likewise, the significance of confidence intervals for mediation hypotheses is interpreted, as discussed below. In particular, the percentile approach is applied to the bootstrap re-sampling with a 95% confidence. The advantage of this approach is that it does not presuppose any distribution of data (Chin, 2010).

The higher-order model (ACAP, innovation and performance as multidimensional constructs) is built by using latent variable scores in Two-Step Approaches (Agarwal and Karahanna, 2000; Chin, 2010; Henseler and Chin, 2010; Wright et al., 2012), as this approach produces more consistent and less biased estimates in the case of small samples than other methods available (Wilson and Henseler, 2007).

Analysis of the control variables fails to reveal any significant paths, hence their exclusion from the model.

In fsQCA, in order to add the values of latent variables in input conditions, arithmetic means of the scores of different indicators of first- and second-order constructs were used. As for the calibration of data, fsQCA Software offers a function with the following structure: *f*_*cod* = *Calibrate*(*cod, full, mid, non*−*full*). *Cod* is the input causal condition for calibration, *f*_*cod* is the fuzzy causal condition, and *full, mid*, and *non-full* are the three values that define the fuzzy set in the calibration process. In the present analysis, the full, mid, and non-full values are 7, 5 and 3, respectively. As for the input condition (*exp*), the function *Expfs* = *Calibrate*(*exp*, 7, 5, 3) has been used, where *fs* is the fuzzy function (Zadeh, 1965; Moreno-Garcia et al., 2014) that was added to the input condition.

### 4.1. Model of simple mediation of absorptive capacity. PLS-SEM results

#### 4.1.1. Analysis of the measurement model

Individual item reliability is appropriate when the factor loading of the item is greater than 0.707, acceptance value recommended by Carmines and Zeller (1979). However, values lower than 0.707 but greater than 0.5 or 0.6 may be considered acceptable (Barclay et al., 1995; Chin, 1998a). Hulland (1999) suggests that factor loadings are also acceptable from a minimum value of 0.4. In this study, indicators and reflective dimensions in Models A and B satisfy this requirement (Tables 3A,B), where the vast majority of values are well-above 0.7. Loadings lower than 0.7 are kept in measurement scales due to their contribution to content validity (Hair et al., 2011; Roldán and Sánchez-Franco, 2012) and because they are positive values above the threshold value of 0.5. In addition, as will be seen below, loadings do not affect composite reliability, extracted variance or discriminant validity of the construct, which reinforces the decision to keep these indicators in the models (Camisón and Villar-López, 2010).

**Table 3:** Overview of survey items: loadings, construct reliability and convergent validity for the measurement models.

**Code**	**Model A**				**Model B**				**Model C**			
	**λ**	**α**	**ρ**	**AVE**	**λ**	**α**	**ρ**	**AVE**	**λ**	**α**	**ρ**	**AVE**
CONDF		0.8783	0.9117	0.6749		0.8783	0.9121	0.6762		0.8783	0.9121	0.6764
CONDFQ1	0.8794				0.8776				0.8800			
CONDFQ2	0.8227				0.8364				0.8339			
CONDFQ3	0.7052				0.6884				0.6896			
CONDFQ4	0.8544				0.8589				0.8566			
CONDFQ5	0.8349				0.8366				0.8384			
DORG		0.7444	0.8835	0.7916		0.7827	0.9000	0.8184		0.7992	0.9083	0.8320
DORGEC	0.8481	0.9359	0.9508	0.7957	0.8750	0.9359	0.9517	0.7982	0.8985	0.9359	0.9517	0.7981
DORGQ1	0.9428				0.9225				0.9151			
DORGQ2	0.9571				0.9430				0.9373			
DORGQ3	0.9397				0.9282				0.9263			
DORGQ4	0.8486				0.8721				0.8803			
DORGQ5	0.7552				0.7928				0.8011			
DORGS	0.9295	0.8778	0.9154	0.324	0.9334	0.8778	0.9171	0.7357	0.9256	0.8778	0.9167	0.7344
DORGQ6	0.7040				0.7507				0.7784			
DORGQ7	0.8463				0.8354				0.8156			
DORGQ8	0.9152				0.8986				0.8918			
DORGQ9	0.9381				0.9349				0.9333			
ACAP						0.7533	0.8849	0.6602		0.7524	0.8840	0.6580
AD					0.6617	0.7117	0.8083	0.5937	0.6735	0.7117	0.7973	0.5810
ADQ1					0.8651				0.8772			
ADQ2					0.8599				0.8571			
ADQ3					0.5416				0.4890			
AS					0.8483	0.8621	0.9072	0.7108	0.8271	0.8621	0.9072	0.7108
ASQ1					0.8851				0.8867			
ASQ2					0.9039				0.9007			
ASQ3					0.8341				0.8323			
ASQ4					0.7397				0.7435			
TRANSF					0.8363	0.9540	0.9668	0.8793	0.8351	0.9540	0.9669	0.8795
TRANSFQ1					0.8940				0.8884			
TRANSFQ2					0.9537				0.9549			
TRANSFQ3					0.9321				0.9359			
TRANSFQ4					0.9694				0.9702			
EX					0.8855	0.8896	0.9316	0.8203	0.8927	0.8896	0.9308	0.8186
EXQ1					0.8128				0.7979			
EXQ2					0.9441				0.9504			
EXQ3					0.9534				0.9569			
INN										0,9521	0,9765	0,9542
INNTEC									0.9741	0.9380	0.9529	0.8018
INNQ1									0.9109			
INNQ2									0.8949			
INNQ3									0.8823			
INNQ4									0.9343			
INNQ5									0.8527			
INNADMIN									0.9795	0.9136	0.9393	0.7945
INNQ6									0.8858			
INNQ7									0.8728			
INNQ8									0.9201			
INNQ9									0.8861			

*λ, Loading; α, Cronbach's alpha: ρ, Composite reliability; AVE, Average variance extracted; CONDF, Training (reflective construct); DORG, Organizational performance (superordinate multidimensional construct); DORGEC, Economic performance; DORGS, Satisfaction performance; ACAP, absorptive capacity (superordinate construct); AD, Acquisition; AS, Assimilation; TRANSF, Transformation; EX, Exploitation; INN, Innovative capacity (superordinate construct); INNTEC, Product innovation; INNADMIN, Process innovation*.

Construct reliability is evaluated using Cronbach's alpha (α) and composite reliability (ρ). Both indices aim to measure the internal consistency of a construct, although the use of composite reliability is more appropriate in PLS, as it is a higher measurement than Cronbach's alpha (Fornell and Larcker, 1981). For both indices, 0.7 is the basic point of reference (Nunnally, 1978; Fornell and Larcker, 1981; Nunnally and Bernstein, 1994). All reflective constructs and dimensions in this study are reliable, as most of them present values greater than 0.8 (strict reliability) (Tables 3A,B).

The average variance extracted (AVE) measures convergent validity, i.e., if all indicators represent the same latent variable. All constructs and reflective dimensions reach convergent validity, exceeding the threshold value of 0.5 (Fornell and Larcker, 1981) (Tables 3A,B).

Finally, Table 4 shows evaluation results of discriminant validity (degree to which a construct differs from others). For each of the constructs, the square root of AVE exceeds correlations between constructs (Fornell and Larcker, 1981), and construct loadings are higher in their respective constructs than in cross-loadings (Barclay et al., 1995). This proves the discriminant validity of the measures used.

**Table 4:** Inter-construct correlations matrix: discriminant validity (Model B).

	**1. ACAP**	**2. CONDF**	**3. DORG**
1. Absorptive capacity (ACAP)	0.8125		
2. Training (CONDF)	0.7737	0.8223	
3. Organizational performance (DORG)	0.7320	0.5329	0.9047

*Diagonal elements are the square root of the variance shared between the constructs and their measures (average variance extracted). Off-diagonal elements are the correlations among constructs. For discriminant validity, diagonal elements should be larger than off-diagonal elements*.

#### 4.1.2. Evaluation of the structural model

Following the confirmation of convergent validity, discriminant validity and reliability of the measurement model, testing of the relationships between variables takes place. In order to determine the different effects and test mediation, this study follows the steps proposed by Hair et al. (2014), who in turn follow the steps that Preacher and Hayes (2004, 2008) propose.

**Figure 2:**
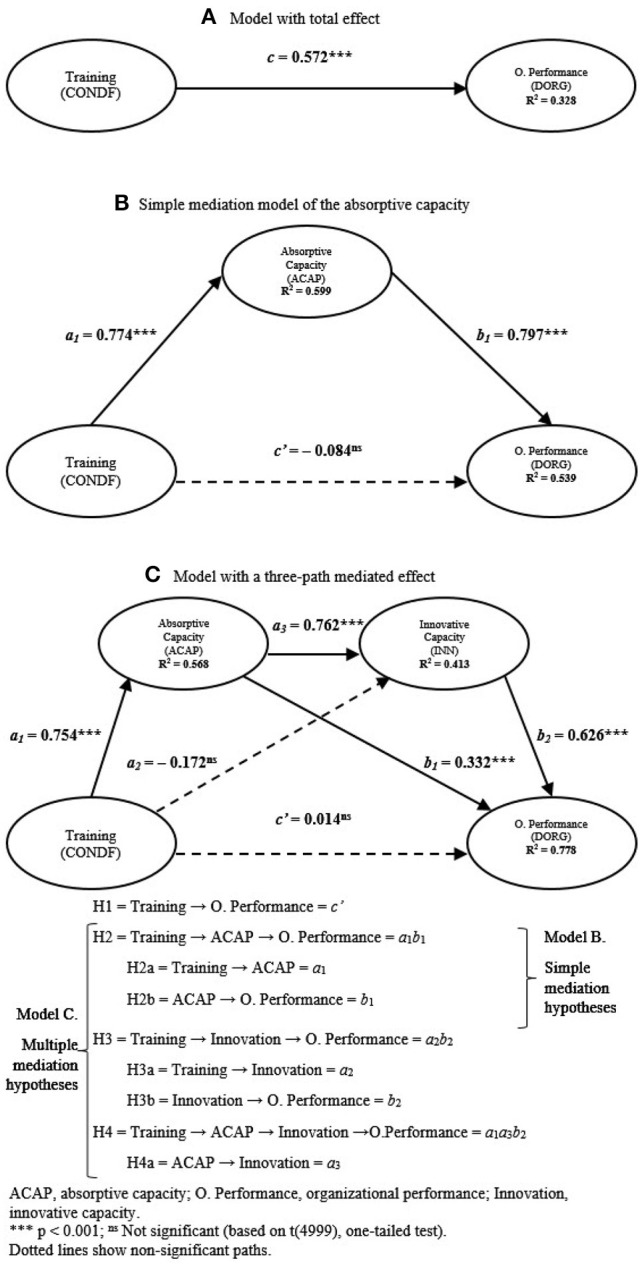
Structural models results: simple and three-path mediation models. **(A)** Model with total effect. **(B)** Simple mediation model of the absorptive capacity. **(C)** Model with a three-path mediated effect.

The first step is checking the direct effect, which should be significant if the mediator is not included in the model (c) (Model A, Figure 2A). While this is not a necessary condition (Zhao et al., 2010), it makes analysis much easier to understand and interpret (Hair et al., 2014). As discussed earlier, the significance test is performed through the bootstrapping procedure (5,000 subsamples). In the study, the overall effect of training (CONDF) on performance (DORG) is positive and significant (β = 0.572; *t*-value surpasses the minimum level indicated by Student's t-distribution with one tail and *n*−1 degrees of freedom, where *n* is the number of subsamples with a 99.9% confidence level. Therefore, the probability of being wrong in rejecting the hypothesis is null: *p* < 0.001. This result is reinforced by applying the percentile method on bootstrap re-sampling in a 95% confidence interval (Table 5A).

**Table 5:** Structural equation models results.

**T.I.E. (M. A)**	**Model B**						**Model C**					
**D.E**.	**β**	**P.E**.	***t*****-value**	**Percentile 95%**		**Sup**.	**β**	**P.E**.	***t*****-value**	**Percentile 95%**		**Sup**.
				**Lower**	**Upper**					**Lower**	**Upper**	
TET. O. P.	0.5723^***^		8.3753	0.4439	0.7151	Yes	0.5723^***^		8.3753	0.4439	0.7151	Yes
H1	−0.0836^ns^		0.6174	−0.3278	0.2025	Not	0.0137^ns^		0.2048	−0.1248	0.1446	Not
TIT. O. P.		0,6164		0.4659	0.7522	Yes		0.5020		0.3724	0.6214	Yes
H2		0.6164		0.4659	0.7522	Yes		0.2500		0.1282	0.3863	Yes
H2a	0.7737^***^		21.4803	0.7003	0.8416	Yes	0.7538^***^		16.9407	0.6583	0.8335	Yes
H2b	0.7967^***^		7.8783	0.5721	0.9753	Yes	0.3317^***^		3.7760	0.1676	0.5083	Yes
H3								-0,1075		−0.2475	0.0690	Not
H3a							−0.1718^ns^		1.3211	−0.4038	0.1095	Not
H3b							0.6255^***^		13.5622	0.5291	0.7101	Yes
H4								0.3594		0.2611	0.4497	Yes
H4a							0.7622^***^		8.5545	0.5549	0.9047	Yes

*Causal relationships: total, direct and indirect effects. T.I.E. (M. A), Total and Indirect Effects/mediation hypotheses (Model A); D.E., Direct Effects (Hypothesis H1 and Sub-hypotheses); β, path coefficient; P.E., Point Estimate; Sup, Supported; TET. O.P., Total Effect of Training on Organizational Performance; TIT. O.P., Total Indirect Effect of Training on Organizational Performance; ACAP, Absorptive capacity*.

*** *p < 0.001; ns Not significant [based on t_(4, 999)_, one-tailed test]*.

*t_(0.05, 4, 999)_ = 1.645158499, t_(0.01, 4, 999)_ = 2.327094067, t_(0.001, 4, 999)_ = 3.091863446*.

The second step is to evaluate the effect of the mediating variable (ACAP) (mediation hypothesis H2). By including the mediator (Model B, Figure 2B, Table 5B), the indirect effect is significant (*H*2 = *a*_1_*b*_1_ = 0.6164, confidence interval does not include zero) and there is a significant (positive) relationship in individual paths that make up this indirect effect: between training and ACAP (H2a = *a*_1_: β = 0.774; *p* < 0.001; confidence interval does not include zero), and between ACAP and performance (H2b = *b*_1_: β = 0.797; *p* < 0.001; confidence interval does not include zero). Now, how much does the mediating variable absorb? To determine the magnitude of this indirect effect, the VAF ratio (Variance Accounted For) (Iacobucci and Duhachek, 2003) indicates the size of the indirect effect in relation to the total effect (direct effect + indirect effect): $VAF=\frac{\left(a_{1}b_{1}\right)}{\left(a_{1}b_{1}+c^{\prime }\right)}$, where the value obtained is greater than 1 (specifically 1.16). After being included in this study, the mediating variable absorbs so much of the positive direct effect (*c*) that it does not only decrease but becomes a negative, not significant effect (*c*′ = −0.084; *p* > 0.05; confidence interval includes zero). Thus, the direct effect disappears: the positive and significant relationship without the mediator (*c*) becomes not significantly negative (*c*′) after including the mediator. A suppressor effect is generated (Hair et al., 2014), characterized by the change of sign of the direct relationship that occurs after including mediating variables. This type of situation always represents full mediation, i.e., absorptive capacity completely mediates the relationship between training and organizational performance. Mediation hypothesis H2 and sub-hypotheses H2a and H2b are supported (Table 5B).

Furthermore, the evaluation of the structural model displays good fit, high consistency, good accuracy and predictive relevance (Table 6B). The model explains 54% of the variance of organizational performance, well above the threshold recommended by literature (Falk and Miller, 1992; Henseler et al., 2009; Hair et al., 2011). All Stone-Geisser *Q*2 blindfolding algorithm values are considerably above zero (ACAP *Q*2: 0.3147; DORG *Q*2: 0.4417), which gives predictive relevance to the model. Likewise, its Goodness-of-Fit criterion (GoF: 0.6241) is solid and confirms its global validation.

**Table 6:** Summary of evaluation of the structural models: consistency, accuracy, predictive relevance, effect sizes and global fit.

**C.: E.E.V**.	**DE^1^**	**E.V**.	***R*^2^ Value**	***Q*2 Value**	**Δ*R*^2^**	***f*2**	**Gof**
**Model A**							0.4900
T. → O.P. = c	Sig		0.3275	0.1768			
**Model B**							0.6241
**Endogenous Latent Variables:**							
**ACAP**			0.5987	0.3147			
H2a = T. → ACAP = *a*_1_	Sig	0.5987					
**O.P. (mediated by ACAP)**			0.5387	0.4417	0.2112	0.4578	
H1 = T. → O.P. = *c*′	Nsig	0.0445^a^					
H2b = ACAP → O.P. = *b*_1_	Sig	0.5832					
**Model C**							0.6654
**Endogenous Latent Variables:**							
**ACAP**			0.5681	0.3001	−0.0306	−0.0708	
H2a = T. → ACAP = *a*_1_	Sig	0.5681					
**Innovation**			0.4130	0.4133			
H3a = T. → Innovation = *a*_2_	Nsig	0.0692^b^					
H4a = ACAP → Innovation = *a*_3_	Sig	0.4822					
**O.P. (mediated by ACAP and Inn.)**			0.7777	0.6487	0.2390	1.0751	
H1 = T. → O.P. = *c*′	Nsig	0.0070					
H2b = ACAP → O.P. = *b*_1_	Sig	0.2447					
H3b = Inn. → O.P. = *b*_2_	Sig	0.5260					
**Improvement of Model C over Model A**					0.4502	2.0252	

C.: E.E.V., Comparison of Models: Effects on Endogenous Variables; DE, Direct Effects; E.V., Explained Variance; T., Training; O.P., Organizational Performance; Inn., Innovation

1 *Results from Table 5: Sig. denotes a significant direct effect; Nsig. denotes a non-significant direct effect*.

a *This sum (0.0446 + 0.5832) is not equal to R^2^ (0.5387), note that the contribution of training to the explained variance of O. Performance is negative, but very small. This commonly occurs when the sign of the zero-order correlation is the opposite of the sign of the path coefficient (Menard, 2009). In Model B, the path coefficient c′ is negative, but very small and non-significant direct effect*.

b *This sum (0.0692 + 0.4822) is not equal to R^2^ (0.4130), note that the contribution of training to the explained variance of Innovation is negative, but very small. In Model C, the path coefficient a 2 is negative, but very small and non-significant direct effect*.

### 4.2. Model with a three-path mediated effect (Model C). PLS-SEM results

#### 4.2.1. Analysis of the measurement model

Tables 3C, 7 show the parameters associated with the evaluation of the measurement model for Model C.

**Table 7:** Inter-construct correlations matrix: discriminant validity (Model C).

	**1. ACAP**	**2. CONDF**	**3. DORG**	**4. INN**
1. Absorptive capacity (ACAP)	0.8112			
2. Training (CONDF)	0.7538	0.8224		
3. Organizational performance (DORG)	0.7378	0.5156	0.9121	
4. Innovative capacity (INN)	0.6327	0.4027	0.8409	0.9768

*Diagonal elements are the square root of the variance shared between the constructs and their measures (average variance extracted). Off-diagonal elements are the correlations among constructs. For discriminant validity, diagonal elements should be larger than off-diagonal elements*.

All indicators and reflective dimensions surpass the strictest cut-off point of 0.707, except for items *condfq3* and *adq3s* and the Acquisition Dimension (AD), which are very close to this value and, in any case, exceed the minimum value of acceptance, so they are kept in the model for the same reasons discussed above in relation to Model B. Cronbach's alpha α and composite reliability ρ of all reflective constructs and dimensions reach the threshold value of 0.7. In fact, most of them present greater values of strict reliability than 0.8. Likewise, the AVE of all reflective constructs and dimensions surpass the recommended value of 0.5. Thus, the resulting values support reliability and convergent validity of the reflective scales under study (Table 3C).

Finally, to ensure discriminant validity, correlations between each pair of constructs are checked to ensure they do not exceed the value of the AVE square root in each construct (Table 7). In addition, factor loadings are higher in their respective constructs than in cross-loadings.

#### 4.2.2. Evaluation of the structural model

The bootstrapping procedure (5000 subsamples) is applied together with the percentile approach on bootstrap re-sampling with a 95% confidence in order to generate the bootstrap confidence intervals of beta weights and indirect effects for mediation hypotheses. Four out of six direct effects described in Figure 2C are significant (*p* < 0.001; the confidence intervals did not include zero). The analysis of these results show that hypotheses H1 and H3a are not supported (Table 5C: direct effects). The direct effect of training on performance when mediating variables are included (*c*′) is no longer significant (*c*′ = 0.014; *p* > 0.05; the confidence interval includes zero). Likewise, the direct effect of training on innovative capacity is negative, albeit very low and not significant (β = 0.172; *p* > 0.05; the confidence interval includes zero).

The model presents an appropriate predictive power for all dependent variables (Table 6C). So much so that organizational performance reaches the highest *R*^2^ value (0.78). Of all immediate relationships received by performance, its *R*^2^ is achieved through the variance explained partly via ACAP (24.5%) and partly via innovation (52.6%). The relationship via training is not significant and only explains 0.7% of the variance in performance, well below the minimum threshold proposed by Falk and Miller (1992). On its part, innovative capacity explains 48.2% via ACAP, whereas the contribution of training on the explained variance of innovation is negative and very low. This commonly occurs when the sign of the zero-order correlation is opposite to the sign of the path coefficient (Menard, 2009). Indeed, as noted above, β is negative, very low and not significant (−0.172). The model is also evaluated using the cross-validated redundancy index (*Q*2). Results are shown in Table 6C, confirming that the structural model has a satisfactory predictive relevance for the three endogenous variables: dynamic capabilities of absorption and innovation and performance. Additionally, its overall Goodness-of-Fit criterion (*GoF*:0.6654) is very good.

To test mediation hypotheses (H2, H3 and H4), the study applied the analytical approach described by Preacher and Hayes (2008) and Taylor et al. (2008). This approach is followed in recent studies by authors such as Castro and Roldán (2013). Indirect effects are specified and contrasted against mediators. The study also examines the total (c) and direct (H1: *c*′) effects of the independent variable (i.e., training) on independent variable (i.e., performance) (Table 5C). Following Williams and MacKinnon (2008), the bootstrapping procedure was chosen to test indirect effects, as mentioned above. Chin (2010) proposes a two-step bootstrapping procedure to test mediation in PLS: (1) use the model in question including both direct and indirect paths, run a particular bootstrap re-sampling N and explicitly calculate the product of direct paths that make up the indirect path that is being evaluated; (2) estimate the significance using the percentile bootstrap or bias-corrected bootstrap, which has proven to generate confidence intervals less biased and more able to detect non-null effects (Williams and MacKinnon, 2008). This generates a confidence interval of 95% for mediators: ACAP (H2), innovative capacity (H3), ACAP and innovation (H4). When the interval for a mediation hypothesis does not include zero, the indirect effect is significantly different from zero with 95% confidence. As already shown, training has a significant overall effect (c) (Model A, Figure 2A) on performance. When mediators (Figure 2C) are introduced, training has no longer a significant direct effect on performance (H1: *c*′). This means that the dynamic capabilities of absorption and innovation completely mediate the influence of training on performance (Baron and Kenny, 1986). Indeed, as noted above, H1 is not supported. Similarly, H3a is not supported either, which leads to reject mediation hypothesis H3 (causal-steps approach) (Taylor et al., 2008). This situation is also supported in view of results in Table 5C, where the product of β coefficients in the indirect path is negative, very low and not significant (H3: *a*_2_*b*_2_ = −0.11: confidence interval includes zero). However, H2 and H4 are supported, which means that two of the three indirect effects of training on performance included in this research model are significant. Therefore, analyses show that ACAP positively mediates the relationship between training and performance (H2: *a*_1_*b*_1_). Results also show that training is positively associated with higher dynamic capabilities of absorption and innovation, connected with higher levels of business performance (H4: *a*_1_*a*_3_*b*_2_): this is the most important indirect effect found. Finally, innovative capacity does not mediate the relationship between training and performance by itself—i.e., not in a simple mediation via innovation (H3: *a*_2_*b*_2_)—but it sequentially does so preceded by ACAP in a double mediation via ACAP + Innovation (H4, three-path mediated effect).

### 4.3. Results from the comparison of Models A, B, and C

This section analyzes and compares research models based on Table 6 (results from PLS-SEM).

Table 6 summarizes relationships and direct effects on endogenous variables in each of the models and results from the evaluation of the three models through the PLS method.

Firstly, this table shows significant results of direct effects transferred from evidence shown in Table 5. Next, it shows explained variances of endogenous variables (*R*^2^), along with the contributions of the direct effects of each of their immediate antecedent variables or predictor variables to these explained variances. *R*^2^ indicates the amount of variance of the construct that is explained by the model. Although Falk and Miller (1992) set at 0.1 the critical value of *R*^2^, levels from 0.19, 0.33 and 0.67 are considered weak, moderate and substantial levels, respectively (Chin, 1998b). For comparison purposes, it is worth observing changes in the *R*^2^ indicator, to determine whether the influence of a particular latent variable on a dependent construct has a substantial impact. These *R*^2^ changes are calculated based on the relevance of the effect (*f*2) (Chin, 1998b). Likewise, *Q*2 indicators of endogenous constructs are reported. When these indicators are greater than zero, they indicate predictive relevance of the structural model for that particular construct. The GoF criterion represents a measure of overall fit of the PLS path model, which ranges between 0 and 1. Although there is no consensus on a cut-off value, (i.e., the greater than 0, the better fit), Wetzels et al. (2009) establish the following reference values for the fit size to comprehensively validate the model: GoFsmall = 0.1, GoFmedium = 0.25, GoFlarge = 0.36, in line with the effect sizes recommended for *R*^2^ by Cohen (1988) (small: 0.02; medium: 0.13; large: 0.26). Similarly, 0.02, 0.15, and 0.35 *f*2 levels can be considered as indicating a small, medium or large effect (respectively) of a latent predictor variable at the structural level (Chin, 1998b).

These *f*2 indices, along with *Q*2 test and GoF criterion, allow comparing the different alternative models A, B, and C.

In view of the above results, the first observation is as follows: in general, based on an initial model with acceptable values (Model A), all parameters progressively improve (i.e., increase) from Model A to B, from B to C and therefore, from A to C, which is the most important increase. In other words, Model B is better than Model A, and Model C is better than Model B and much better than Model A. Not only Model C is the best model, but it also presents excellent values of structural assessment, as discussed below.

All three models present high levels of *R*^2^ for all endogenous variables, where performance reaches the highest value (substantial) in Model C. The same applies to *Q*2 values of the dependent variable (performance): all *Q*2 values are greater than 0, where Model C reaches the highest value, thus becoming the model with the greatest predictive relevance for performance. And the same applies to GoF values, which are higher than 0.36 (cut-off value for large *R*^2^ effect sizes) in the three models, where Model C is the model that presents the best overall fit. When the model does not include mediators (Model A), *R*^2^ (0.33), *Q*2 (0.18) and GoF (0.49) are acceptable. When the mediating variable ACAP (Model B) is included, the explained variance of performance increases from 33 to 53.9%, with a wide significance of the effect (*f*2 = 0.46), which involves a significant improvement. *Q*2 values of performance also improve, increasing from 0.18 to 0.44, as well as GoF values, increasing from 0.49 to 0.62. When including the two mediating variables (ACAP and innovative capacity) (Model C), *Q*2 values of performance increase from 0.44 to 0.65, as well as GoF: 0.62 to 0.67. When including the second mediating variable (innovative capacity), ACAP transfers a part of its direct effect to performance through innovative capacity. This explains the greater amount of performance variance than before including innovative capacity. Regarding the explained variance of ACAP on performance, it decreases from 58.3% in Model B to 24.5% in Model C, although innovative capacity emerges here as a new net contribution (52.6%), much higher than the explained variance of performance. Similarly, although the *R*^2^ of the ACAP in Model C is slightly smaller than that in Model B, it does not yield a significant *f*2, implying that the importance of this decrease is small and insignificant. In contrast, the explained variance of performance increases from 53.9% to 77.8%, with a very wide significance of the effect (*f*2 = 1.08), which involves a substantial improvement.

Based on the above, the significant improvement of Model C on Model B is confirmed. Therefore, it dramatically improves Model A too, by substantially improving *Q*2 of performance, GoF and finally, the explained variance of performance in more than 45 percentage points, with a substantial impact (*f*2 = 2.03).

### 4.4. Configurational and causal analysis using fsQCA approach

fsQCA was run in order to generate the combinations of conditions leading to *dorgfs*. fsQCA calculates three solutions: the complex, the intermediate, and the parsimonious solutions. Following the recommendations of Ragin (Ragin, 2008, Chapter 11), the parsimonious and intermediate solutions will be used in this test (Table 8). As it can be seen, both solutions obtain the same combinations of conditions. The obtained model is informative since its consistency is above 0.74 (Woodside, 2013).

**Table 8:** Results from fsQCA (truth tables).

**Causal configuration**	**RC**	**UC**	**C**
**RESULTS OF THE PARSIMONIOUS SOLUTION (OUTCOME: dorgfs)**			
Condffs^*^~acapfs^*^~innfs	0.383915	0.061503	0.812987
~Condffs^*^~acapfs^*^innfs	0.389346	0.087787	0.861240
Condffs^*^acapfs^*^innfs	0.646224	0.302435	0.858273
Solution coverage: 0.806904			
Solution consistency: 0.794239			
**RESULTS OF THE INTERMEDIATE SOLUTION (OUTCOME: dorgfs)**			
Condffs^*^~acapfs^*^~innfs	0.383915	0.061503	0.812987
~Condffs^*^~acapfs^*^innfs	0.389346	0.087787	0.861240
Condffs^*^acapfs^*^innfs	0.646224	0.302435	0.858273
Solution coverage: 0.806904			
Solution consistency: 0.794239			

*RC, Raw coverage; UC, unique coverage; C, consistency; dorg, organizational performance; Condf, training; acap, absorptive capacity; inn, innovation; -fs, fuzzy function*.

Table 9 shows two obtained solutions using the notation introduced by Ragin (2008). We want to emphasize that the parsimonious and the intermediate solutions are identical. For this reason, there are no conditions of the contributing causal condition type, neither present nor absent, in that table. The combinations from *A*_1_ to *A*_3_ have been obtained. *A*_3_ reaches the maximum raw coverage and a great unique coverage (0.30). The other two combinations (*A*_1_ and *A*_2_) attain 0.06 and 0.09 as unique coverage. *A*_1_ corresponds with the hypothesis H1, and shows that there exists a relationship between the variable condffs and the outcome in absence of the mediators. This relationship gets a low value of unique coverage that indicates a weak influence of the training in the organizational performance. *A*_2_ reaches a value of unique coverage greater than *A*_1_, which shows that innovation by itself has a greater influence than training on the organizational performance. Finally, *A*_3_ is the more prominent combination to achieve a strong performance. It gets a value more than three times greater than the other two combinations, showing a big influence in the organizational performance. This last combination indicates that the three conditions are needed to achieve good organizational performance.

**Table 9:** Configurations for achieving a good organizational performance.

	**Solution**		
	***A*_1_**	***A*_2_**	***A*_3_**
Condffs	●	⊗	●
Acapfs	⊗	⊗	●
Innfs	⊗	●	●
Raw coverage	0.383915	0.389346	0.646224
Unique coverage	0.061503	0.087787	0.302435
Consistency	0.812987	0.861240	0.858273

*● = Core causal condition (present)*.

*⊗ = Core causal condition (absent)*.

## 5. Discussion

This study aims to deepen the relationship between training and performance, unraveling the process by which training is transferred to performance. Such process consists of a set of complex interconnections, some of which are indirect, thus not directly perceived by individuals. The study examines the role of absorptive capacity both as a mechanism to identify and translate external inputs of knowledge from training into tangible benefits, and as a means to reach innovation. In turn, both ACAP and innovation influence performance. This finding represents a way for firms to capitalize the efforts they make in training their employees. Likewise, dynamic capabilities explain why firms that make the same efforts in training get different training results.

This vision of training based on dynamic capabilities gives rise to define the concept of Dynamic Training as the organizational knowledge flows resulted from training, which align or adjust to firms absorptive and innovative capacities in order to mobilize these capacities toward improving organizational performance.

### 5.1. Implications for research and theory

Results from conducting both methodologies (PLS-SEM and fsQCA) in the study of the same phenomenon validate and corroborate the suggested conceptual model. Results are convergent and more refined thanks to the contribution of each of these methods. This methodological dualism is already in itself a significant contribution to this research study.

Another novelty of this study is its contribution to literature in the field of absorptive and innovative capacities, by identifying a new role of absorptive capacity, as well as understanding the antecedent variables of innovative capacity that are related to training and their effects on organizational performance. Absorptive capacity fulfils the essential input function of innovative capacity. Therefore, a formal training model is suggested, with explanatory and predictive capacity, thus solving the gap in literature, which has systematically defended the positive relationship between training and business performance, but has failed to agree on an explanatory and predictive model regarding how that relationship occurs.

### 5.2. Implications for firms practice

The most important conclusion of this study is that training translates into results if the dynamic capabilities of absorption and innovation mediate this process. Therefore, when firms plan their design of training programs, they should mobilize these dynamic capabilities and assess their level of current development, in order to decide the appropriate setting allocated to each dimension.

PLS-SEM extends the model to include absorptive and innovative capacities, thus obtaining the “true” relationship between training and performance. This relationship is systematically affected by the afore mentioned capacities, which in turn are explained by training. The fsQCA supports that organizational performance outcomes are explained by the configurations in a holistic causality fashion, rather than through any single condition. For example, we can infer that a certain “recipe” (i.e., *A*_3_) results in a good organizational performance, but we cannot infer that training (or absorptive capacity or innovation) independently exerts a positive influence on the organizational performance. As a consequence, we can conclude that the three conditions are important to reach a good organizational performance highlighting the innovation among them. This conclusion supports the conclusions achieved with PLS. Therefore, training based on dynamic capabilities will guide and ease the design of appropriate human resources strategies, which will transform training into results. From all the absorptive capacities, realized capacity are necessary for potential capacities to be realized.

These findings have the following important implications for human resources managers:

1. Firms should establish training plans that consider absorptive capacity needs, not just training needs.
2. Absorptive capacity offers firms a means of appropriation of training outcomes, thus reducing the risk of retaining trained employees.
3. Training encourages the development of dynamic capabilities (Teece et al., 1997), particularly, absorptive capacities that will eventually influence innovation.
4. For training to translate into results, firms must: train employees (both permanent employees and new recruits), promote the development of their skills, make training applicable to the job they perform and keep them updated on the last changes in the firm. While all these conditions are still necessary, they are not sufficient, so firms should pay special attention to absorptive and innovative capacities.
5. To foster absorptive capabilities, in line with previous studies such as the work conducted by Flatten et al. (2011) or Jansen et al. (2005), training programs should incorporate both training activities and organizational activities; for example, with Service Level Agreements, comprehensive communication plans (DirCom), or the use of intranet technologies and enterprise social software and workgroup collaboration web-based tools for the development of dynamic capabilities.

Finally, some limitations of this study are related to common problems of the dominant logic in research (Rong and Wilkinson, 2011; Woodside, 2011, 2013), cross-sectional surveys, one-shot, self-reports, Likert-scales, multiple regression analysis (MRA) techniques and structural equation models (SEM). Although the application of the fsQCA methodology has helped to alleviate these problems (Woodside, 2013), the following considerations for future research may be done.

Training may have delayed effects over time (D'Arcimoles, 1997; Murray and Raffaele, 1997). The mediation effect would need time to develop (training would increase absorptive capacity over time, which would increase future performance, again over time). In the same way, innovation may have delayed effects over time (Laplagne and Bensted, 1999; Kor and Mahoney, 2005; Kostopoulos et al., 2011; Dostie, 2014). However, two aspects must be pointed out: the low influence of the training effort made in a period on the results in the next period (Aragón-Sánchez et al., 2003), and that most empirical studies of the relationship between innovation and results are cross-sectional in nature (Hall, 2011), as is the case with most studies on training and results.

To explore this issue and reduce the common method bias, it would be advisable to carry out a time-lagged (multi-wave) data collection. One way is to create a temporal separation by introducing a time lag between the measurement of the predictor (for example, from actual data) and criterion variables (from a new data set), without compromising anonymity. This is achieved by using a linking variable that is not related to the respondents identity. This would be possible thanks to the support of the LimeSurvey software.

## Author contributions

All authors listed, have made substantial, direct and intellectual contribution to the work, and approved it for publication.

### Conflict of interest statement

The authors declare that the research was conducted in the absence of any commercial or financial relationships that could be construed as a potential conflict of interest. The reviewer TS and handling Editor declared their shared affiliation

## Appendix

### Measurement items for constructs

CONSTRUCT/dimension/indicator code

**Training (CONDF) (reflective construct)** *Training conditions*. Please respond to the following statements honestly and in line with your own opinion. To answer use the following criteria by rating your agreement on the scale: 1 = never, 2 = very rarely, 3 = rarely, 4 = sometimes, 5 = often, 6 = very often, 7 = always.

**– CONDFQ1** The firm trains its workers.
**– CONDFQ2** The firm promotes the development of skills of its workers.
**– CONDFQ3** The training that workers receive from the organization is applicable to the job.
**– CONDFQ4** The organization updates employees about changes that occur in it.
**– CONDFQ5** When a new worker joins, he or she receives instruction about the company (availability of training for new hires).

**Organizational performance (DORG) (superordinate multidimensional construct, reflective second-order construct or molecular second-order factor)** Evaluate your firms performance as compared to the average of your competitors on the scale, where 1 equals much worse and 7 much better: *Economic performance (DORGEC) (reflective first-order dimension)*

**– DORGQ1** Mean economic profitability (pre-interest and pre-tax profits/total net assets).
**– DORGQ2** Mean financial profitability (after-tax profits/own funds).
**– DORGQ3** Mean sales profitability (pre-interest and pre-tax profits/sales).
**– DORGQ4** Annual sales growth.
**– DORGQ5** Market share gain.

Satisfaction performance (DORGS) (reflective first-order dimension)

**– DORGQ6** Labor productivity.
**– DORGQ7** Customers satisfaction.
**– DORGQ8** Others stakeholders satisfaction.
**– DORGQ9** Strength of competitive position.

**Absorptive Capacity (ACAP) (Superordinate Construct)**

*Acquisition (AD) (reflective first-order dimension)* Please specify to what extent your company uses external resources to obtain information (e.g., personal networks, consultants, seminars, internet, database, professional journals, academic publications, market research, regulations, and laws concerning environment/technique/health/security):

**– ADQ1** The search for relevant information concerning our industry is every-day business in our company.
**– ADQ2** Our management motivates the employees to use information sources within our industry.
**– ADQ3** Our management expects that the employees deal with information beyond our industry.

*Assimilation (AS) (reflective first-order dimension)* Please rate to what extent the following statements fit the communication structure in your company:

**– ASQ1** In our company ideas and concepts are communicated cross-departmental.
**– ASQ2** Our management emphasizes cross-departmental support to solve problems.
**– ASQ3** In our company there is a quick information flow, e.g., if a business unit obtains important information it communicates this information promptly to all other business units or departments.
**– ASQ4** Our management demands periodical cross-departmental meetings to interchange new developments, problems, and achievements.

*Transformation (TRANSF) (reflective first-order dimension)* Please specify to what extent the following statements fit the knowledge processing in your company:

**– TRANSFQ1** Our employees have the ability to structure and to use collected knowledge.
**– TRANSFQ2** Our employees are used to absorb new knowledge as well as to prepare it for further purposes and to make it available.
**– TRANSFQ3** Our employees successfully link existing knowledge with new insights.
**– TRANSFQ4** Our employees are able to apply new knowledge in their practical work.

*Exploitation (EX) (reflective first-order dimension)* Please specify to what extent the following statements fit the commercial exploitation of new knowledge in your company (NB: Please think about all company divisions such as R&D, production, marketing, and accounting):

**– EXQ1** Our management supports the development of prototypes.
**– EXQ2** Our company regularly reconsiders technologies and adapts them accordant to new knowledge.
**– EXQ3** Our company has the ability to work more effective by adopting new technologies.

**Innovative Capacity (INN) (Superordinate Construct)** Please tick the number that best reflect how your organization has been doing so far relative to the major competitors in your industry (1 = Worst in industry, 7 = Best in industry):

Product innovation (INNTEC) (reflective first-order dimension)

**– INNQ1** The level of newness (novelty) of our firms new products.
**– INNQ2** The use of latest technological innovations in our new products.
**– INNQ3** The speed of our new product development.
**– INNQ4** The number of new products our firm has introduced to the market.
**– INNQ5** The number of our new products that is first-to-market (early market entrants).

Process innovation (INNADMIN) (reflective first-order dimension)

**– INNQ6** The technological competitiveness of our company.
**– INNQ7** The speed with which we adopt the latest technological innovations in our processes.
**– INNQ8** The updated-ness or novelty of the technology used in our processes.
**– INNQ9** The rate of change in our processes, techniques and technology.
